# Under-Water Gold Mining in Georgia

**Published:** 1881-10

**Authors:** 


### UNDER-WATER GOLD MINING IN GEORGIA.

The cheapness with which large amounts of earth in river-beds can be washed for gold by the new process of under-water hydraulic mining is awakening great expectations from the owners of river rights in Northern Georgia. Hitherto the cost of mining in the ordinary way has made the working of these streams comparatively unprofitable. By the new process it is claimed that the beds of the Chestattee and Chattahoochee Rivers cannot fail to yield abundantly. The Georgia State Geologist reports that two companies have been formed for prosecuting this work, using boats of the International Vacuum Dredging Boat Company. The first boat, now under construction at Dahlonega, is expected to begin work the middle of September. These boats cost from $6,000 to $10,000 each. Many miles of the rivers named have been leased for working, the price named being ten per cent. of the yield.—*Scientific American.*

